# Incidence of fraud and adulterations in ASEAN food/feed exports: A 20-year analysis of RASFF’s notifications

**DOI:** 10.1371/journal.pone.0259298

**Published:** 2021-11-05

**Authors:** Iyiola Oluwakemi Owolabi, Joshua Akinlolu Olayinka

**Affiliations:** 1 School of Food Science and Technology, Faculty of Science and Technology, Thammasat University, Khong Luang, Thailand; 2 Logistics Analytics and Supply Chain Management Program, International College, Walailak University, Nakhon Si Thammarat, Thailand; Cairo University, EGYPT

## Abstract

This paper explored the occurrence of food fraud and adulterations (FFA) in exports from the Association of South- East Asia Nations (ASEAN), with implications on food chain and international trade. Data from European Union Rapid Alert System for Food and Feed (EU RASFF) about FFA notifications on ASEAN exports for a period of 20 years (2000–2020) were extracted and analyzed. Results from this study revealed that of all ten ASEAN member countries, seven had cases of FFA notified in the database with Thailand (n = 47, 32%) and the Philippines (n = 37, 26%) receiving the highest frequency of notifications in the region. There was a statistical significance difference in frequency of notifications received on products from these seven countries with herbs and spices ranking highest (n = 22, 15%). Highest notifications of FFA on ASEAN exports came from the United Kingdom (n = 31, 21%). All the seven countries experienced border rejections and consequent destruction of food products especially on exports from Indonesia where 95% of product with FFA were border rejected. Border rejections on products from these countries were significantly different. Therefore, a thorough implementation system, appropriate testing and constantly updating each country’s FFA database could aid actions in curtailing future events.

## Introduction

The conventional idea of food safety is based on the accessibility of food products that are without hazards. However, recent trends may alter this concept by incorporating the authenticity of food products as a vital component in validating food safety. Legally, food adulterations include an evident violation of fair-trade principles and caution, while defrauding the consumers who are unable to receive the products (whether in quantity or quality) for which they have paid for [1]. Therefore, food fraud and adulterations (FFA) are important facts that cause serious setbacks to food safety through neglecting the origin, composition, and adverse effects of food adulterations on consumers’ health. By definition, FFA is regarded as an intentional substitution, addition, tampering or misrepresentation of food or food ingredients, food packaging or misguiding information on a product to achieve economic gain [2]. Food fraud and adulteration has been an ancient and exploitative criminal practice over the years [3]. Nonetheless, through the past decades, the issue of food fraud has appeared as a significant hazard in various countries due to direct consequences on public health and international trade. Therefore, adulterated foods pose a serious threat to international standards by endangering the health of consumers through various side effects on human [4]. Food fraud and adulteration could have a direct impact on health of consumers. For instance, addition of melamine to infant formula [5], lead to turmeric powder [6], toxic chemicals to milk [7], formaldehyde in fish and unreported allergens added to food products [8], and injection of shrimps with gel for them to look larger [9]. On the other hand, the health risk could be indirect, especially when the nutritional standard of a food product is compromised due to the use of inferior ingredients, thereby robbing the consumer of the intended health benefits. Food fraud and adulteration has increasingly gained so much attention because of the great quantity of food produced, imported, and exported globally. Hence, the deprivation of confidence by customers, investors, and concerned authorities due to FFA occurrences can be a lot more detrimental than the direct impact on the economy.

The Association of Southeast Asian Nations (ASEAN) established on the 8^th^ of August 1967 in Bangkok, Thailand, consists of 10 member countries, namely Indonesia, Malaysia, Philippines, Singapore, Thailand, Brunei Darussalam, Viet Nam, Lao PDR, and Myanmar [10]. This community was founded to accelerate the economic growth and to collaborate efficiently for significant utilization of their industries and agriculture, expansion of trade, evaluation of problems related to international trade, improving the standards of their people, amongst others. There is generally lack of sufficient information and accurate data on FFA in ASEAN and in Asia at large. However, this region is still known to be at high risk from FFA regarding human health, consumer confidence and international trade [11]. The global supply of food products, coupled with long, complicated, and continually difficult-to-track chains, absence of traceability and transparency, have generated novel opportunities for FFA and its implication on food safety and supply chain [12]. As there are few to none published reviews and studies, including national reports on FFA in this part of the world, the European Commission still stands as a valuable source of food fraud cases on food products exported from ASEAN over the years. Hence, this paper examines a 20-year occurrence of FFA in food products from ASEAN and analyze the impact on the supply chain management and international trade in this community. In addition, recommendations and alternative strategies are proposed to reduce the incidences contingent on the establishment of methods in detecting FFA, good supply chain management and effective tracking systems.

### Global food fraud

**Fig 1:**
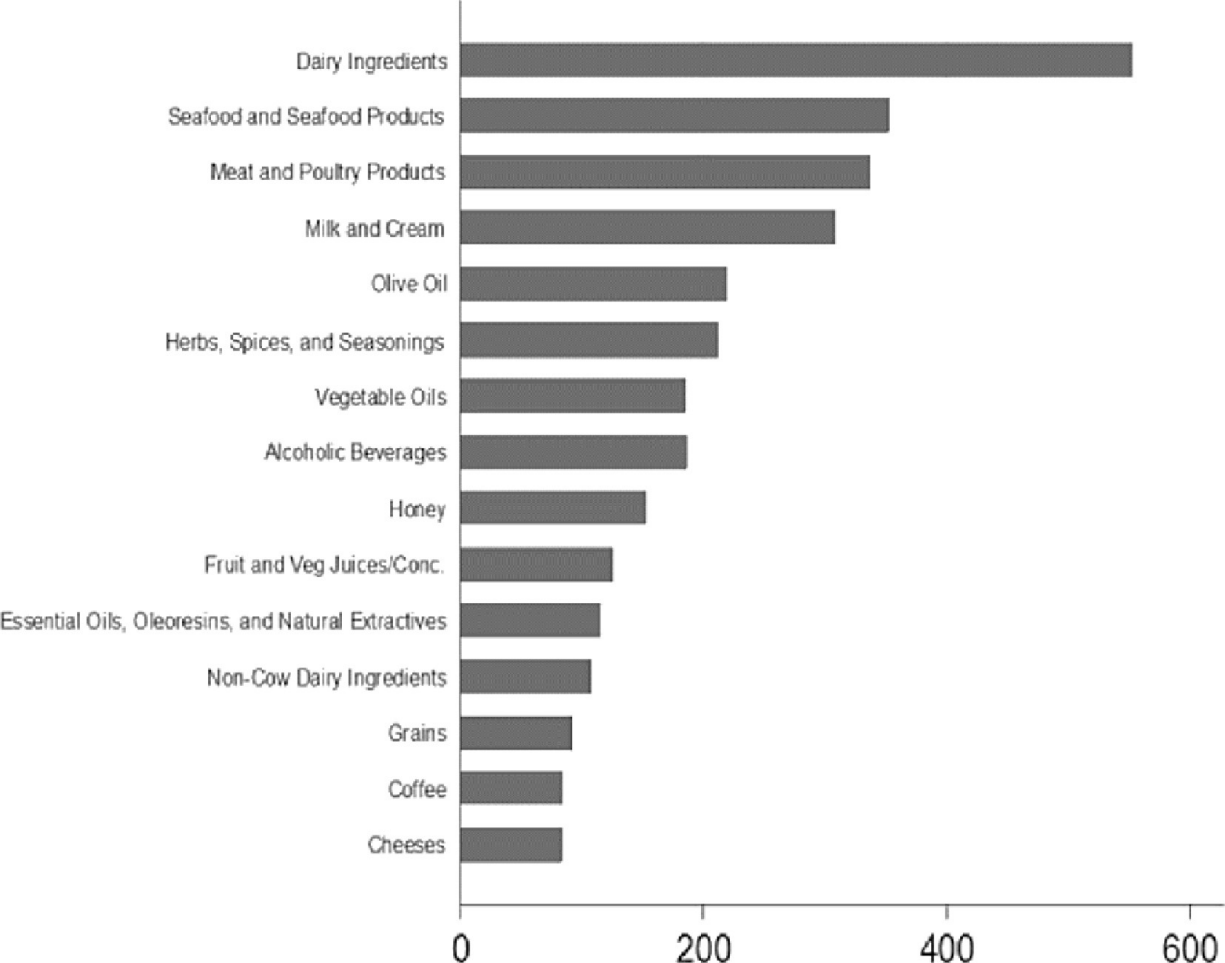
Commodities with the highest number of notifications in the global food fraud. Database Source: [16]; extracted from USP Food Fraud Database (https://decernis.com/solutions/food-fraud-database/).

Food fraud, including its subcategory of “economically motivated adulteration” has generally been considered as a compelling economic issue occurring worldwide decades ago [13]. Nonetheless, current incidents have proven that this unwholesome practice not only affects businesses and economies (as it is often accompanied by loss of sales, rejection, or destruction of food products), but also the confidence of the consumers. While there is no precise information on the global prevalence of food fraud, the Consumer Brands Association (CBA), formerly known as the Grocery Manufacturers Association (GMA) predicted that food fraud may cost between $10 billion and $15 billion loss to the global food industry, therefore affecting about 10% of all food products sold commercially [14]. In the absence of the ability to distinguish counterfeit high quality food products from the authentic quality ones, the health of the consumer could be at risk. Food fraud started as a lucrative opportunity, by a way of decreasing the primary ingredients in food products for additional profits. The whole concept of global food fraud cannot be completely understood, partly because of the deceitful nature of the practice and due to flaws in tracking, monitoring, and reporting systems. Generally, detection of food products because of potential fraud across the supply chain, if undistributed to consumers, are managed on a business-business basis without reporting such cases publicly [15]. Some commodities are specifically considered to be mostly subjected to fraud including honey, herbs and spices, meats, dairy products, seafood, and olive oil. As revealed in Fig 1, the food product with the highest number of notifications in the Decernis Food Fraud Database is dairy ingredients, then seafood and meat/poultry products, respectively. Although information for dietetic foods, dietary supplements and fortified foods are unavailable in this Food Fraud database, it is important to note that this food product category has high vulnerability to food fraud.

However, the European Commission’s Rapid Alert System for Food and Feed (RASFF) provides a more detailed data on food fraud/adulterations for countries over the years. RASFF was created in 1979 by EU member states with the intention to promptly share information on health risk associated with food and feed. EU member states are legally obliged to report information that concerns direct or indirect health risk from food and feed. RASFF database, therefore, contains information such as reported incidence with type and date of notification, reason for notification, country of origin, notifying country, and risk decision among others.

## Materials and methods

Data was obtained online from the RASSF portal [17]. Search criteria for global FFA notifications from RASFF starting from January 1, 2000, to December 31, 2020 (20 years). All “food and feed” product categories were included, while “adulteration/fraud” was selected in the Hazard category. Then, names of each member state in ASEAN were entered as keyword in the search with the “notification”, “type”, and “commodity group” columns left unselected to include all notifications relating product categories. Data from the RASFF website for notification from ASEAN countries (N = 145) were exported directly to Microsoft excel 2020 (Microsoft 365 MSO) for generation descriptive statistics and further analysis. Statistics of frequency, percentage, z-statistics, and chi-square were used to analyze data. Individual notification included data such as product category, date, reference, product type, notification type, notification basis, notified by, countries concerned, subject, action taken, distribution status, and risk decision. In cases where two countries (for example, one country for product origin, while the second for processing and/or packaging) were mentioned for a raw food/feed product, the country of origin for the notified raw product was considered. Data from year 2000 to 2002 and year 2005 were unavailable and therefore excluded from analysis. Moreover, it is important to note that there may be underreporting of FFA in RASFF, however, it is the most comprehensive database for notification on FFA publicly available. RASFF system report food safety relevant incidences but is not the information tool to transmit food fraud information among EU member states. The Administrative Assistance and Cooperation was established to transmit food fraud information among EU member states in confidence through AAC-FF system and the information is not publicly available. The tables reported in this manuscript capture cases which have also a food safety tangent like missing document, use of non-authorized additives, illegal trades etc. which are relevant information that could be useful within ARASFF in ensuring that safe food and feed products that meet the standard regulations are exported from the ASEAN member states in the future.

Based on the description made available in the “subject” section of RASFF notifications, the data were categorized by the authors as presented in Table 1.

**Table 1:** Categories of FFA notifications in RASFF database.

Fraud and adulteration type	Data description
Health certificate(HC)/analytical report	Missing, absent, improper, or fraudulent health certificates or analytical report
Illegal importation	Unauthorized or illegal import, transit, or trade
Tampering	Tampering, fraud, or adulteration
Common entry document (CED)	Fraudulent, expired, missing or improper CED, analytical report or import declaration
Expiration date (ED)	Expiration date
Mislabeling of product	Mislabeling

## Results and discussion

### Frequency of notifications

During the period of 2000 to 2020, a total of 1641 original notifications for FFA were logged on the RASFF database, of which 145 notifications (8.8%) were involved in products from the ASEAN member countries. Nonetheless, it should be noted that the notifications from RASFF may cause an overestimation of notifications of FFA incidents as the same product may receive more than one notification for different fraud/adulteration cases. In addition, some non-compliance product may be detected after distribution in the markets of several EU member states [18]. At the end of the search, only 7 out of 10 countries had “adulterations/fraud” notifications, which were extracted for the period of 20 years. To aid the classification of the list of various products within EU RASFF, 19 food product categories and 1 feed product category (feed materials) were identified. Countries with available FFA data include Thailand, Indonesia, Malaysia, Myanmar, Philippines, Singapore, and Viet Nam.

**Fig 2:**
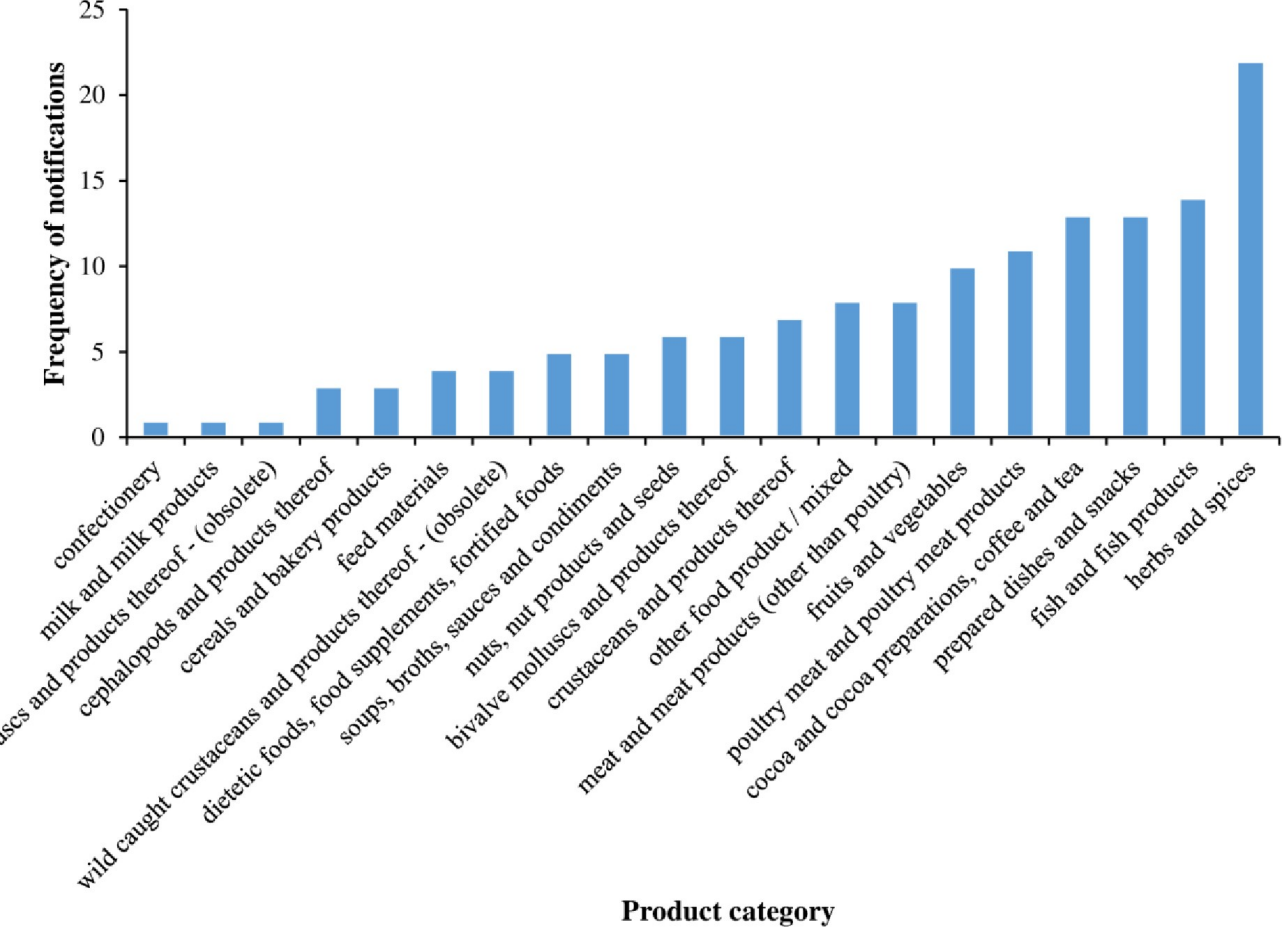
Frequency of food fraud notifications in RASFF by food/feed product category the year (2000–2020).

**Fig 3:**
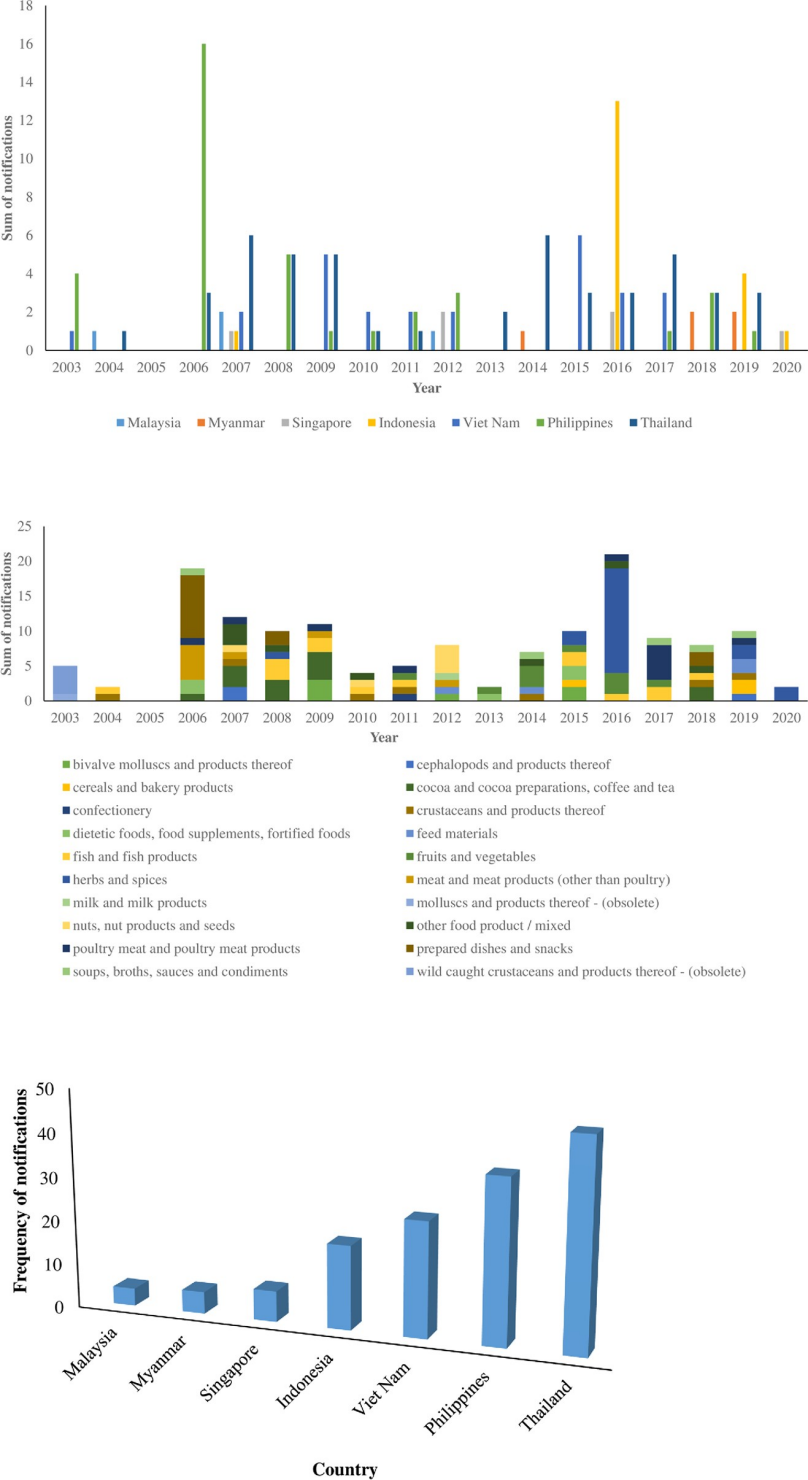
a. Distribution of food fraud notifications in RASFF by year in ASEAN countries (2000 and 2020). b. Distribution of food fraud notifications in RASFF by product category in ASEA countries (2000 and 2020). c. Number of food fraud notifications in RASFF by country (2000 and 2020).

The summary of all food and feed products categories notified on ASEAN exports between 2000 and 2020 are displayed in Fig 2. Of the total of 145 notification for FFA on ASEAN food/feed products, the top 5 products with highest frequency of notification were found with herbs and spices (n = 22, 15.2%), followed by fish and fish products (n = 14, 9.7%), prepared dishes and snacks (n = 13, 9%), cocoa and cocoa preparations, coffee, and tea (n = 13, 9%), poultry meat and poultry meat products (n = 11, 7.6%), fruits and vegetables (n = 10, 6.9%), respectively. However, each of other product categories (15 food categories) recorded less than 10 notifications throughout for the member countries. To check if the product category significantly differs by observed frequency and not due to chance, chi-square test statistics was used and the result was χ^2^ = 74.45, *p < 0*.*000*. This shows that these products have significantly different frequency, and these observed frequencies did not happen by chance. Evaluating the frequency of notifications by year (Fig 3A) from 2000 to 2020, FFA notifications were recorded throughout except for 2000, 2001, 2002, 2003, and 2005 where there were no fraud/adulteration notifications on any food/feed products.

Remarkably high incidence of FFA were reported in 2006 (19 notifications) and 2016 (21 notifications), while lowest values for FFA were recorded in 2004, 2013 and 2020. The upsurge in notifications observed in these years could be due to the higher frequency of notifications received especially on prepared dishes and snacks from Philippines (n = 13) in 2006 and herbs and spices from Indonesia (n = 16) in 2016, respectively (Fig 3B). The number of notifications for Philippines in 2013 and Indonesia in 2016 were significantly higher at z = 3.46 *p <0*.*001* and z = 4.51 *p<0*.*001* respectively. In other words, these notifications were significantly different from other notifications and could not have happen by chance.

The ASEAN Rapid Alert System for Food and Feed (ARASFF) was founded in December 2006 as a bilateral pilot project between the Royal Thai Government and the European Union and was later extended to other member states in ASEAN [19]. This system was developed as a tool to rapidly covey information on newly recorded risks of unsafe or adulterated food/feed products with action taken to prevent the items from reaching the consumers. The absence of notification for some of the years could be attributed to the lack of adequate traceability system for especially high-risk products and inability of ASEAN member states to provide extensive report on food/feed products exported from their countries at both within ASEAN and international level. From the FFA notifications by country (Fig 3C), food/feed products exported from Thailand received the highest number of notifications (47), followed by Philippines (37). Least frequency of notifications was reported on products exported from Myanmar (5) and Malaysia (4) over the period of 20 years.

**Fig 4:**
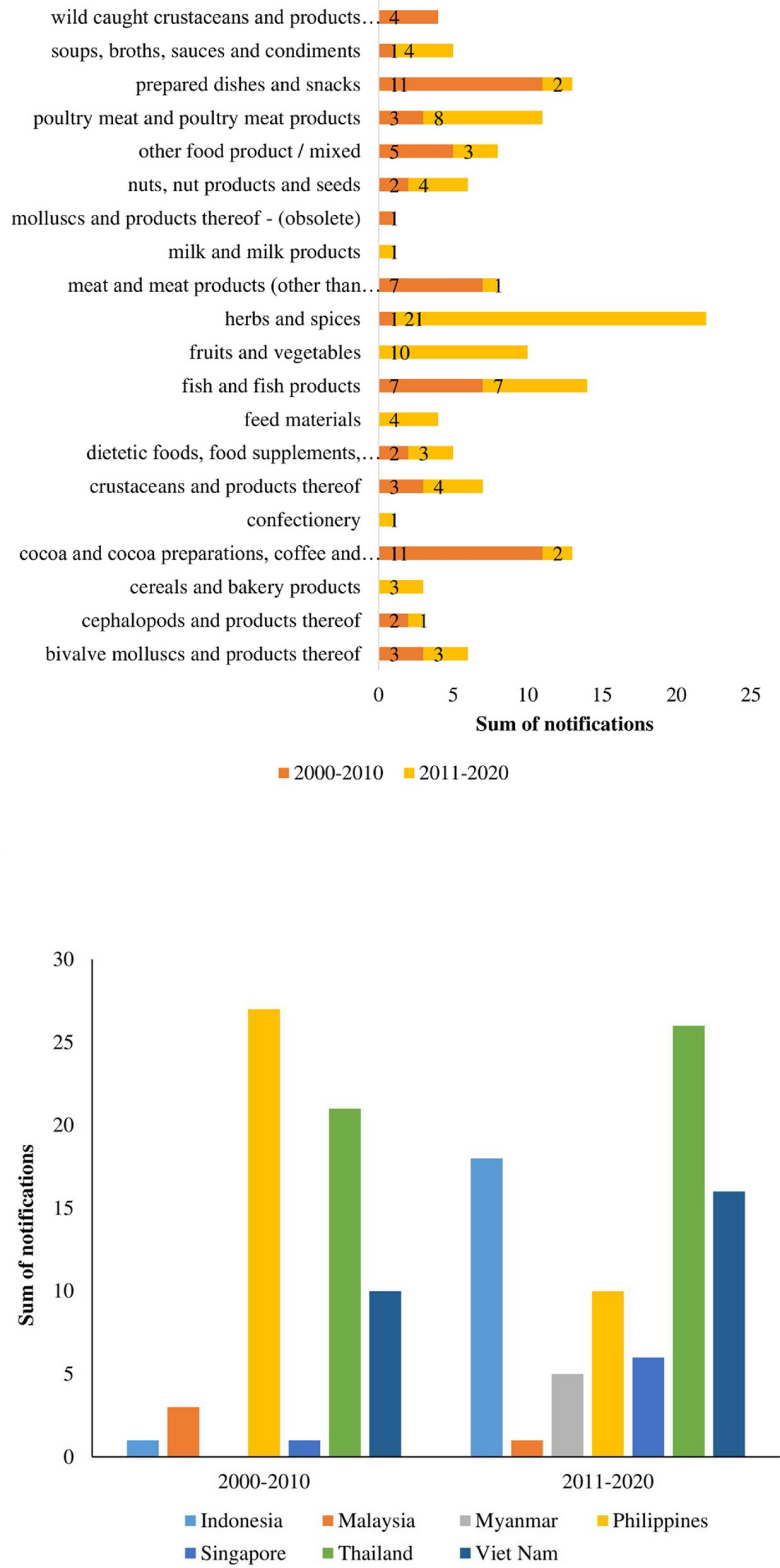
a. Distribution of food fraud notifications in RASFF by decade and by product category (2000 and 2020). b. Distribution of food fraud notifications in RASFF by decade and by country (2000 and 2020).

Comparing by decade (Fig 4A and 4B), the herbs and spices again, contributed to an increase in FFA notifications in the second decade (2011–2020), while products such as fruits and vegetables, feed materials, and cereals and bakery products were notified for FFA only in the second decade. In addition, all products from the 7 ASEAN member countries recorded incidences of FFA notifications in RASFF except for products from Myanmar which had no record of FFA in the first decade. From the sum of all notifications by decade, FFA incidences rose from 43% in the first decade to 57% in the second decade, which may indicate increase in food/feed exports from ASEAN, more product testing and extra rigorous monitoring or regulatory standard in the importing countries.

The overview of fraud/adulterations in food products exported from the ASEAN member states are presented in Table 2.

**Table 2:** Overview of FFA in food/feed products from ASEAN (200–2020).

**THAILAND**											
**Product type**	**Food**	**cephalopods and products thereof**	**cereals and bakery products**	**cocoa and cocoa preparations, coffee, and tea**							
	**Year**	**2007**	**2015**	**2006**	**2007**		**2008**		**2009**		
	Subject	attempt to illegally import frozen octopus from Indonesia exported	absence of health certificate(s) for seaweed instant rice noodles	unauthorized placing on the market of herbal tea containing Senna leaves from Thailand	unauthorized placing on the market herbal tea containing 53.57% senna leaves; 17.86% senna fruit and 28.57% chrysanthemum	unauthorized placing on the market herbal tea containing 75% Senna leaves and 25% Senna fruits	unauthorized placing on the market of herbal infusion containing Garcinia, Senna leaves and pods	unauthorized placing on the market of herbal infusion containing senna leaves and senna pods	unauthorized placing on the market of herbal tea containing senna leaves, pods and seeds	unauthorized placing on the market of flavored green tea containing Senna and Garcinia	unauthorized placing on the market of herbal infusion containing Senna
**Product type**	**Food**	**crustaceans and products thereof**		**dietetic foods, food supplements, fortified foods**				**fish and fish products**			
	**Year**	**2007**	**2018**	**2006**		**2013**	**2015**	**2004**	**2008**	**2009**	**2015**
	Subject	paraffin presence in shrimp pastes	absence of health certificate(s) for shrimp powder	unauthorized placing on the market of Senna herbal infusion	unauthorized placing on the market of Senna herbal infusion	unauthorized placing on the market of herbal tea containing senna	unauthorized placing on the market of herbal tea containing senna	absence of health certificate(s) for tuna	fraudulent health certificate(s) for frozen whole fish	absence of health certificate(s) for canned tuna fish in vegetable oil	expiry dates changed of fish products
**Product type**	**Food**	**fish and fish products**		**fruits and vegetables**					**herbs and spices**	**nuts, nut products and seeds**	**other food product / mixed**
	**Year**	**2016**	**2018**	**2011**	**2013**	**2014**			**2008**	**2010**	**2007**
	Subject	attempt to illegally import mussels in sauce	unauthorized placing on the market of frozen sushi burger	attempt to illegally import fresh herbs and vegetables from Thailand, via Sweden	unauthorized placing on the market of canned yanang leaves extract	absence of Common Entry Document for coriander leaves, yard long beans, peppermint, eggplants	attempt to illegally import egg plants	attempt to illegally import betel leaves	unauthorized placing on the market of herbal tea containing senna leaves, senna fruits and *Chrysanthemum indicum*	unauthorized placing on the market of dried sliced betel nut	attempt to illegally import various products of animal origin
	**Food**	**other food product / mixed**			**poultry meat and poultry meat products**						**soups, broths, sauces, and condiments**
	**Year**	**2014**	**2016**	**2018**	**2009**	**2016**	**2017**			**2019**	**2014**
	Subject	illegal import of frozen insects and sausages	absence of health certificate(s) for fish products and bivalve molluscs	illegal import of collagen casings from Thailand, with raw material from Spain	expiry dates changed of chicken meat in teriyaki from Thailand relabelled in Denmark	poor temperature control and improper common entry document for frozen prepared chicken meat	attempt to illegally import frozen cooked marinated chicken slices containing yoghurt from Thailand	attempt to illegally import frozen cooked marinated chicken diced breast containing yoghurt from Thailand	attempt to illegally import frozen cooked tikka marinated chicken diced breast containing yoghurt from Thailand	attempt to illegally import frozen steamed cooked marinated chicken diced containing yoghurt from Thailand	improper health certificate(s) for fish sauce
**INDONESIA**										** **	
**Product type**	**Food**	**cephalopods and products**			**herbs and spices**						
	**Year**	**2007**	**2019**		**2016**				**2019**		**2020**
	Subject	attempt to illegally import frozen octopus from Indonesia exported from Thailand	E 316—sodium erythorbate unauthorized in and improper health certificate(s) for frozen boiled cut octopus from Indonesia	improper health certificate(s) for pasteurized crabmeat from Indonesia	absence of health certificate(s) for nutmeg from Indonesia	improper health certificate(s) for ground nutmeg from Indonesia	absence of health certificate(s) for whole nutmeg from Indonesia	improper certified analytical report for whole nutmeg from Indonesia	absence of health certificate(s) for nutmeg from Indonesia	absence of health certificate(s) and of certified analytical report for nutmeg from Indonesia	absence of certified analytical report for nutmeg from Indonesia, dispatched from Singapore
**MALAYSIA**											
**Product type**		**Food**		**cephalopods and products**		**meat and meat products (other than poultry)**		**poultry meat and poultry meat products**		**feed materials**	
		**Year**		**2004**		**2007**		**2007**		**2012**	
		Subject		absence of health certificate(s) for frozen prawns from Malaysia		illegal trade of frozen pork tender loins with falsified Italian health mark, dispatched from Malaysia		bad temperature control of and fraudulent take-over declaration for frozen chicken fillets from Brazil		attempt to illegally import (non-approved third country) fish meal from China	
**MYANMAR**		**Food**									
**Product type Year**				**cereals and bakery products**			**cocoa and cocoa preparations, coffee, and tea**		**crustaceans and products**		
Subject				**2019**			**2018**		**2014**		
				improper certified analytical report for parboiled brown rice from Myanmar			illegal import (milk ingredient) of instant tea-mix from Myanmar		fraudulent health certificate(s) for and prohibited substance chloramphenicol (0.32 μg/kg—ppb) in frozen dried shrimp mix from Myanmar		
**SINGAPORE**											
**Product type**		**Food**		**fish and fish products**		**fruits and vegetables**		**herbs and spices**		**nuts, nut products and seeds**	
	**Year**	**2015**		**2016**		**2020**		**2007**		**2012**	
	Subject	expiry dates changed of fish products from Italy	origin unclear of and absence of health certificate(s) and of certified analytical report for betel leaves from India, via Singapore		absence of certified analytical report for nutmeg from Indonesia, dispatched from Singapore		absence of health certificate(s) and of certified analytical report for peanuts from China, via Singapore		improper health certificate(s) for peanut butter crisp from China, via the Philippines		absence of health certificate(s) for peanut butter from China, via the Philippines

**PHILIPPINES**												
**Product type**	**Food**	**meat and meat products (other than poultry)**					**milk and milk products**	**nuts, nut products and seeds**	**other food product / mixed**		**poultry meat and poultry meat products**	
	**Year**	**2006**	**2012**	**2012**	**2008**		**2010**		**2006**		**2011**	
	Subject	illegal import of bacon spread	illegal import of ham spread	illegal import of canned corned beef	illegal import of meat loaf	illegal import of various meat products	illegal import of milk product	absence/improper health certificate(s) for peanut butter/butter crisp	unauthorized operator and absence of health certificate(s) for food products	unauthorized import of various products of animal origin	illegal import of chicken spread	attempt to illegally import chicken spread
**Product type**	**Food**	**prepared dishes and snacks**										
	**Year**	**2006**									**2008**	
	Subject	illegal import of corned beef with onions	illegal import of various meat products	illegal import of prepared dishes	illegal import of sausages	illegal import of canned meat product	illegal import of liver spread	illegal import of beef loaf	illegal import of instant meals	illegal import of bacon and ham spread	illegal import of canned pork and beans	illegal import of canned pork and beef livers’ pate
**Product type**	**Food**	**prepared dishes and snacks**		**soups, broths, sauces, and condiments**					**wild caught crustaceans and products thereof—(obsolete)**		**fish and fish products**	**confectionery**
	**Year**	**2008**	**2006**	**2017**	**2018**	**2019**	**2003**		**2008**		**2011**	
	Subject	illegal import of instant noodles	illegal import of various snacks	illegal import of chicken noodle soup	attempt to illegally import and unauthorized operator for anchovy sauce	absence of health certificate(s) for fish sauce	illegal import of chicken, ham and bacon spread	fraud with shrimps	fraud with frozen shrimps	absence of health certificate(s) for tuna spread	attempt to illegally import ice cream	
**VIET NAM**												
**Product type**	**Food**	**bivalve molluscs and products**						**crustaceans and products**			**meat and meat products (other than poultry)**	
	**Year**	**2003**	**2009**	**2009**	**2012**	**2010**	**2011**		**2015**		**2012**	
	Subject	Absence of health certificate(s) for squid sausages	fraudulent health certificate(s) for frozen mussel meat IQF	fraudulent health certificate(s) for frozen baby clams	fraudulent health certificate(s) for frozen clams	improper import declaration (product not heat-treated) for frozen cooked clams	improper import declaration (product not heat-treated) for frozen cooked clams	absence of health certificate(s) for frozen raw black tiger shrimps	absence of health certificate(s) for frozen raw black tiger shrimps		unauthorized import of dried pork skin from Vietnam, via Sweden	
**Product type**	**Food**	**dietetic foods, food supplements, fortified foods**						**fish and fish products**			**fruits and vegetables**	
	**Year**	**2015**	**2009**	**2010**	**2011**	**2017**	**2015**		**2016**		**2017**	
		unauthorized placing on the market of food supplements containing Stephania or Chelidonium majus	fraudulent health certificate(s) for pangasius fillets	improper health certificate(s) (mentioned seal was missing) for frozen pangasius fillets	poor temperature control—rupture of the cold chain—of and absence of health certificate(s) for frozen pangasius	illegal import of frozen tempura fish sticks, frozen breaded butterfly prawn, frozen tempura prawn and frozen oriental prawn roll	unauthorized operator and absence of health certificate(s) for anchovy sauce	attempt to illegally import fresh mint	attempt to illegally import coriander		absence of health certificate(s) for dragon fruit	
**Product type**	**Food**	**herbs and spices**	**cocoa and cocoa preparations, coffee, and tea**				**other food product / mixed**					
	**Year**	**2015**	**2015**	**2016**		**2009**		**2007**				
		illegal import of mint	missing import declaration for mint	absence of Common Entry Document (CED) for fresh mint	attempt to illegally import fresh mint	unauthorized placing on the market of herbal infusion containing senna leaves	attempt to illegally import and absence of health certificate(s) for various food products	absence of health certificate(s) and improper health certificate(s) for frozen crustaceans and eel				

### Incidence of fraud/adulteration from RASFF on Food/Feed exports from ASEAN

#### Thailand

From the notifications on Thailand exports, food products in the “cocoa and cocoa preparations, coffee, and tea” category received the highest frequency of notifications between 2006 and 2009. This was majorly due to unauthorized placing of herbal tea/infusion and green tea in market containing Senna leaves, fruits, pods, and Garcinia. Until recently in 2019, poultry meat and poultry meat products were notified for expiry dates, poor temperature control, improper entry documents and attempt to illegally import frozen prepared chicken meat, including cooked and steamed chicken meat marinated with yoghurt from Thailand. Products in the “fish and fish products” (2004–2018) and “fruits and vegetables” (2011–2014) categories were reported for FFA due to absence of/fraudulent health certificates, changes in expiry dates, illegal importations, lack of entry documents and authorized placing of products in the market. The rest of the products were notified on similar reasons as well as due to the presence of paraffin in shrimp pastes. From the cases of fraud/adulteration in 2018 and 2019, it is important to note that products categories such as “crustaceans and products”, “fish and fish products”, “other food product /mixed” and “poultry meat and poultry meat products” may still require a more serious monitoring and safety checks to prevent future occurrences, economic loss, and ensure consumer health safety.

#### Indonesia

Cephalopods and products; herbs and spices exported from Indonesia have been on notification for fraud/adulterations from 2007 until 2020. Of all the ASEAN member states, herb and spices from this country were frequently notified (16 notifications) by importing countries for fraud/adulterations based on absence or improper health certificates and certified analytical reports, especially on nutmegs exported from Indonesia between 2016 and 2020. For products in the “cephalopods and products” category, there were reports on illegal importation of octopus from Indonesia but dispatched from Thailand in 2007. In addition, improper health certificates and presence of E316 Sodium erythorbate was found with boiled cut octopus, including lack of proper health certificates for crabmeat directly exported from Indonesia in 2019.

#### Malaysia, Myanmar, and Singapore

From the information on the EU RASFF database, FFA incidence in food products from Malaysia occurred between 2004 and 2007, ranging from the missing health certificates for exported prawns to illegal trading of frozen pork tender loins with forged Italian mark, bad temperature control and deceitful take-over statements on chicken fillets from Brazil. Furthermore, an attempt to illicitly import fish meal from China (unapproved third country) was recorded in 2012 in the “feed materials” category. Although fraud is a significant issue and a source of risk affecting both the safety of the food and feed, feed fraud is almost overlooked and undermined. However, the feed sector often encounters fraud through the raw materials for feed imported from other countries. While it can pose a risk to human and animal safety, it can also reduce the assurance of intended safety including the integrity of the product, trust of supply chain partners and the consumers [20]. In 2014, cases of fraudulent health certificates were recorded on frozen dried shrimp mix from Myanmar as they were found to contain an illegal substance (chloramphenicol). In the “cocoa and cocoa preparations, coffee, and tea” category, there were illegal importations of some instant tea mix reported in 2018 and inappropriate certified analytical reports for brown rice exportations in 2019. For Singapore, most cases were on nuts, nut products and seeds (2007–2012) because of missing health certificates and certified analytical reports for peanuts, peanut butter, and peanut butter crisp. Also, fish products and betel leaves from Singapore were found with changed expiry dates and absence of health certificates or approved analytical reports. However, herbs and spices are still the major food products with current incidence of fraud as recorded on exported nutmegs in 2020.

#### Philippines

Food products in the “prepared dishes and snacks” category exported from the Philippines received the highest notification for FFA between 2006 and 2008. Products such as corn beef, meat products, sausages, canned meat products, liver spread, bacon and ham spread, canned pork and bean, instant noodles and several snacks were reported for illegal importations. The same case of fraud also goes for the meat and meat products since they are often used as raw materials for the prepared dishes exported from the Philippines. The value of exported frozen bovine meat from Philippines grew by 3.48% in 2019, reaching a total of $40, 000 and 0.794% of the total domestic sales of frozen meat products when compared to those in 2018 [21]. However, there is absence of updated information regarding FFA product in EU RASSF database. Other food categories exported were notified based on unauthorized importation, lack of health certificates and improper health documents. Until recent years (2006–2019), there were still reported cases of attempting to illegally import soups, broths, sauces, and condiments from the Philippines.

#### Viet Nam

Exportation of fish and seafood products is known as the second-highest export earner after palm oil, constituting 15% of the total fish exports in the world. Viet Nam and Thailand respectively have been the third and fourth main fish and seafood exporters in the world since 2014 [22]. Food products in “bivalve molluscs and products” category witnessed highest cases of FFA, followed by fish and fish products, and crustaceans and products between 2003 and 2017. Reasons for notifications on these products include missing/fraudulent health certificates found in squid sausages and mussel meat, illegal importation of frozen whole clams in shell and poor storage temperature control. Also, herbs and spices, fruits and vegetables, and other food products were reported for FFA based on similar reasons.

**Fig 5:**
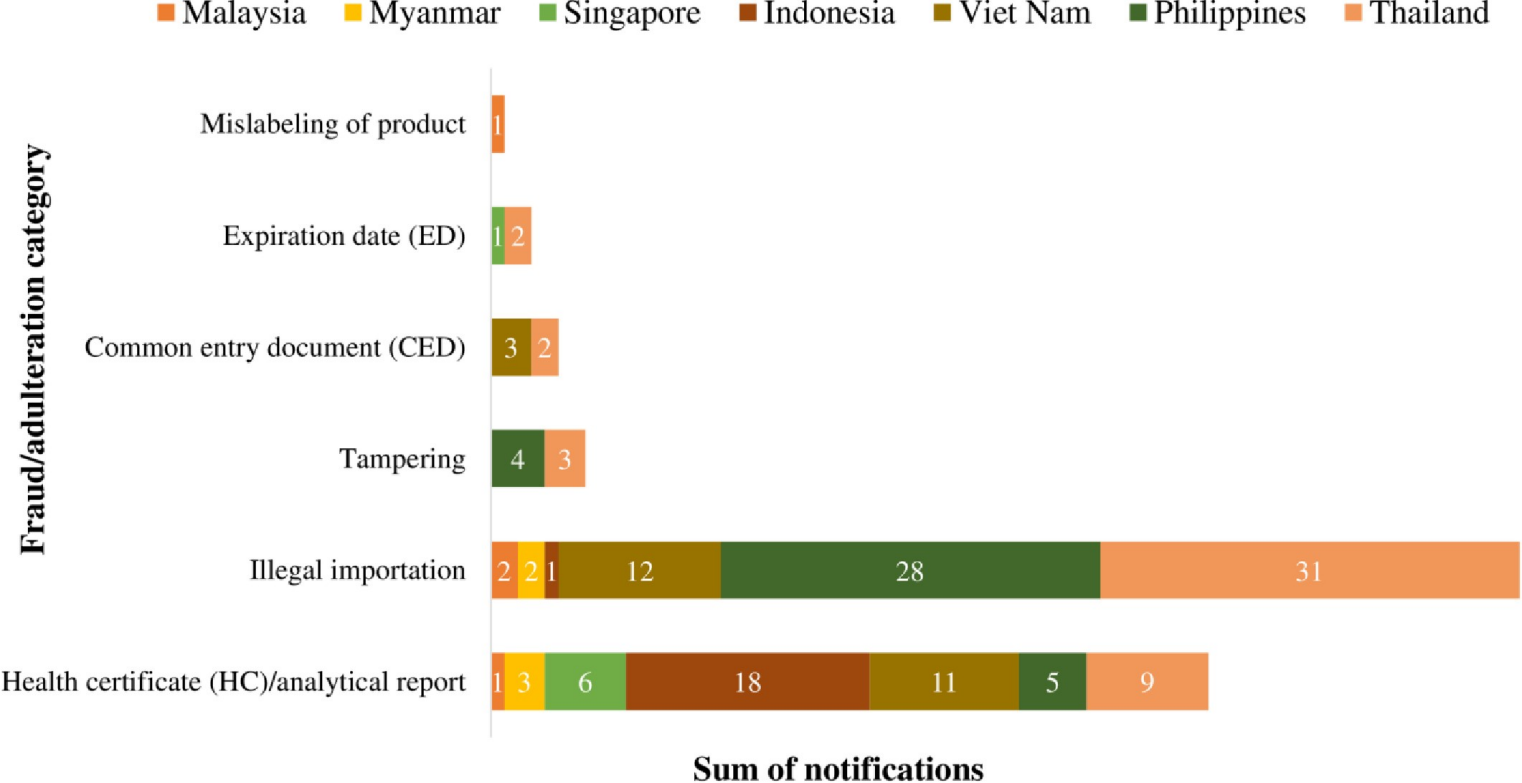
Summary of notifications in RASFF on food/feed product s from ASEAN countries by fraud/adulteration category (2000 and 2020).

To summarize this section, most of the products from ASEAN received FFA notifications based on reasons surrounding illegal importations (most especially Thailand and Philippines), followed by issues on product’s health certificates and analytical reports (Fig 5).

### Impact of food fraud on ASEAN international trade

International trade plays a vital role in relieving the burden of food insecurity and export development through granting producers and consumers market to internationally sell and buy foreign food commodities. By this, farmers or producers from exporting countries can benefit from higher incomes–leading to enhanced food accessibility, availability of increased nutritious diets and improved health outcomes [23]. Although, domestic trade is an important factor for improving food accessibility and availability, food value and supply chain stretches beyond national borders.

**Fig 6:**
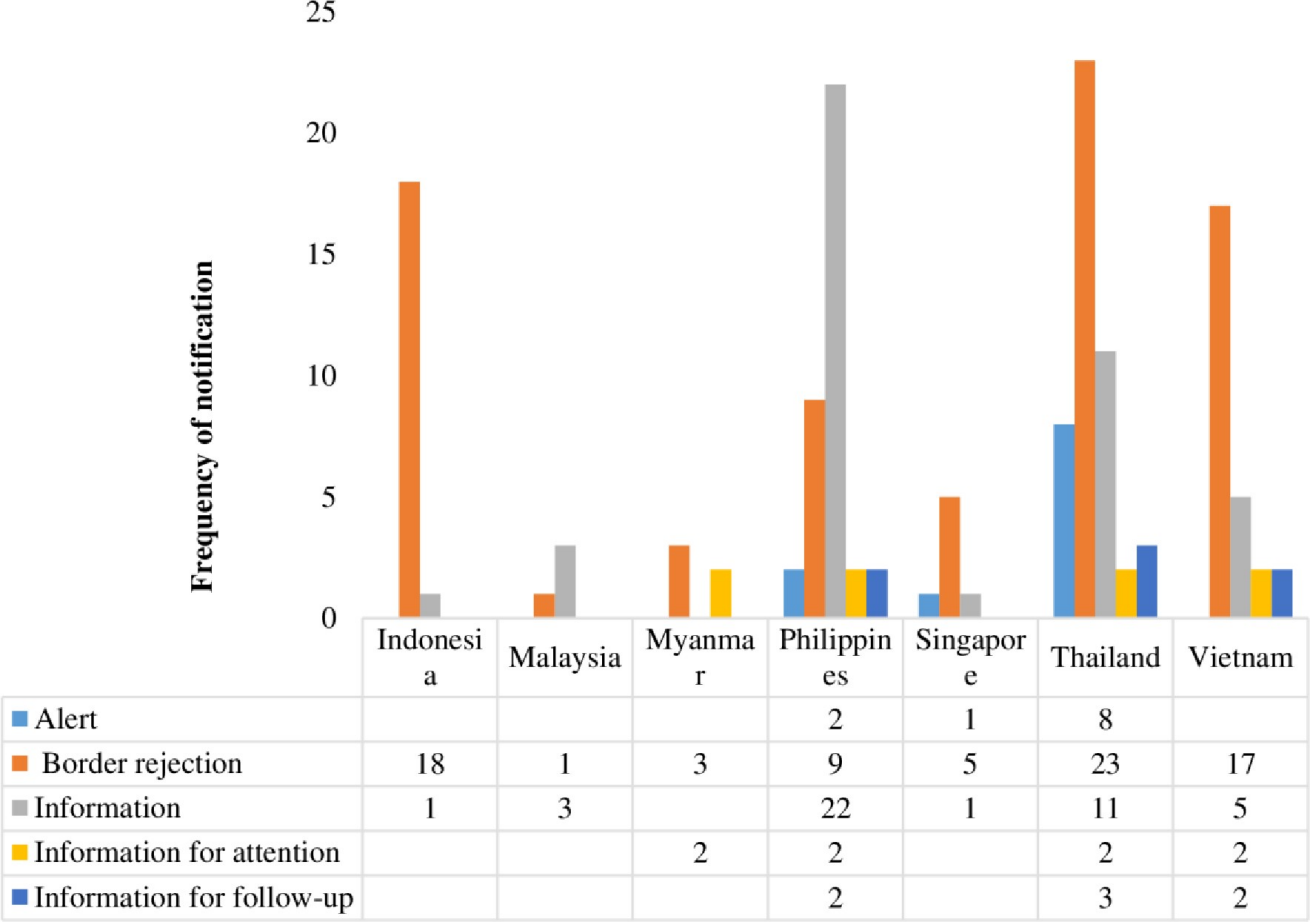
Notification types on food/feed exports from ASEAN (2000–2020).

**Fig 7:**
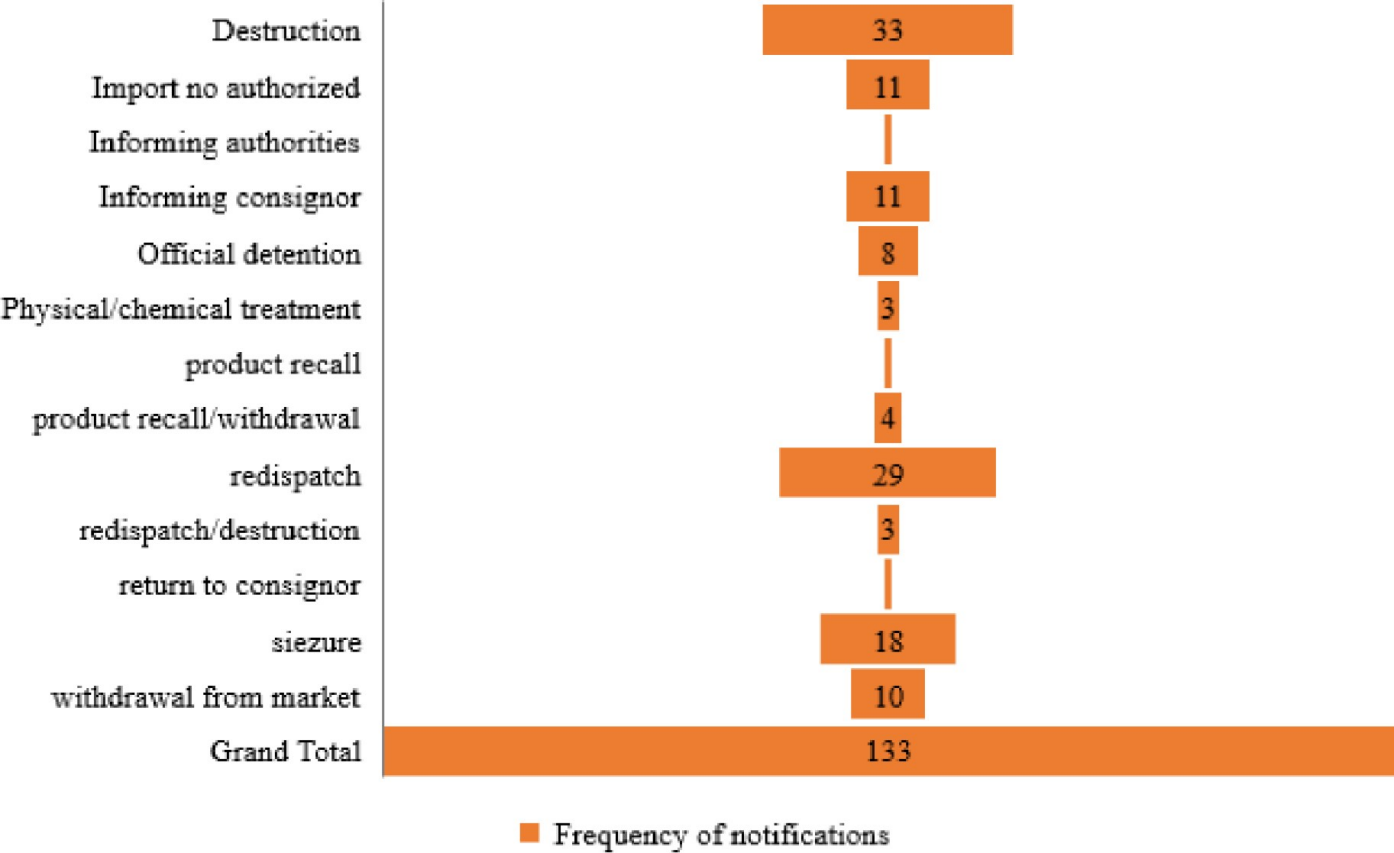
Frequency of actions taken on food/feed exports from ASEAN (2000–2020).

International trade boosts the effective utilization of global limited resources, moreover, trade competition produces more desirable productivity and innovation. Food fraud incidence increases in markets where there are several buyers and sellers across the supply chains. In modern agri-food supply chains, ingredients may be acquired and aggregated into food products through multiple networks of agents and distributors who, themselves have inadequate information of or accountability for the commodities they manage [24]. Trading of food products at an international level may promote fraud possibilities because of absence of preventative legal schemes, comprehensive supply chains, and heightened difficulties in ascertaining the source of fraud [25]. Fig 6 shows the notification types on food/feed products exported from the ASEAN between 2000 and 2020. A larger percentage of the products receiving notifications for FFA were rejected at the border, followed by “information”, “information for attention”, “information for follow up” and “alert”, respectively. Chi-square statistics showed that the observed frequencies of border rejection are significantly different at χ^2^ = 39.87, *p < 0*.*001*. Therefore, border rejections of these countries were statistically different and not due to chance. All the seven ASEAN countries in focus experienced border rejections between 2000 and 2020 with the least occurrence in exports from Malaysia (n = 1). Thailand with highest comparative border rejections (n = 23) since the country’s export has highest notifications among the countries studied. Most of the fraud and adulterations from Indonesia were rejected at border (n = 18, 95%) and these fraud and adulterations occurred mainly in herbs and spices. All herbs and spices from ASEAN countries notified for FFA were mostly (n = 20, 91%) border rejected and majority (n = 16, 73%) of them were exported from Indonesia. Although, incentives from the economy are often present, food fraud may thrive when products are procured from or exported from countries where there are weak or inadequately enforced domestic monitoring or badly structured legal administrations [24]. Unfortunately, 24.81% (33 notifications) of the total food/feed exports with FFA from ASEAN were destroyed, 21.80% (29 notifications) received notifications to redispatch, 13.5% were recorded to be seized, 8.27% received notifications for declined importation, another 8.27% for informing consignor, and 7.52% were reported to be withdrawn from the market, respectively (Fig 7). All other products were subjected to actions such as “official detention”, “physical/chemical treatment”, “product recall/withdrawal”, “return to consignor” and “informing authorities”. Fraud situations could lead to increased cost of international agri-food trade at both public and private levels. The various notification types and actions taken on those products are because of stringent border inspections and testing, consequently leading to increased cost of related food safety assurance [26]. In addition, food fraud promotes buyers and sellers international trade costs. For example, potentially counterfeit product which poses a health risk on humans and animals, especially from a country/region suspected to have greater potential of food safety hazards, may experience extra scrutiny and border delays. These additional cost from international agri-food trading due to fraud may as well cause a rift between fraudulent and authentic product prices [27]. Fig 6 reveals that between 2000 and 2020, food/feed products from ASEAN member states were exported to a total of 22 countries which are mainly European countries. Of all the notifying countries, products exported to the United Kingdom and Italy received the highest notifications (n = 31 and 30, respectively) for FFA. This was followed by Norway (n = 18), Netherlands (n = 16) and Germany (n = 11), respectively.

**Fig 8:**
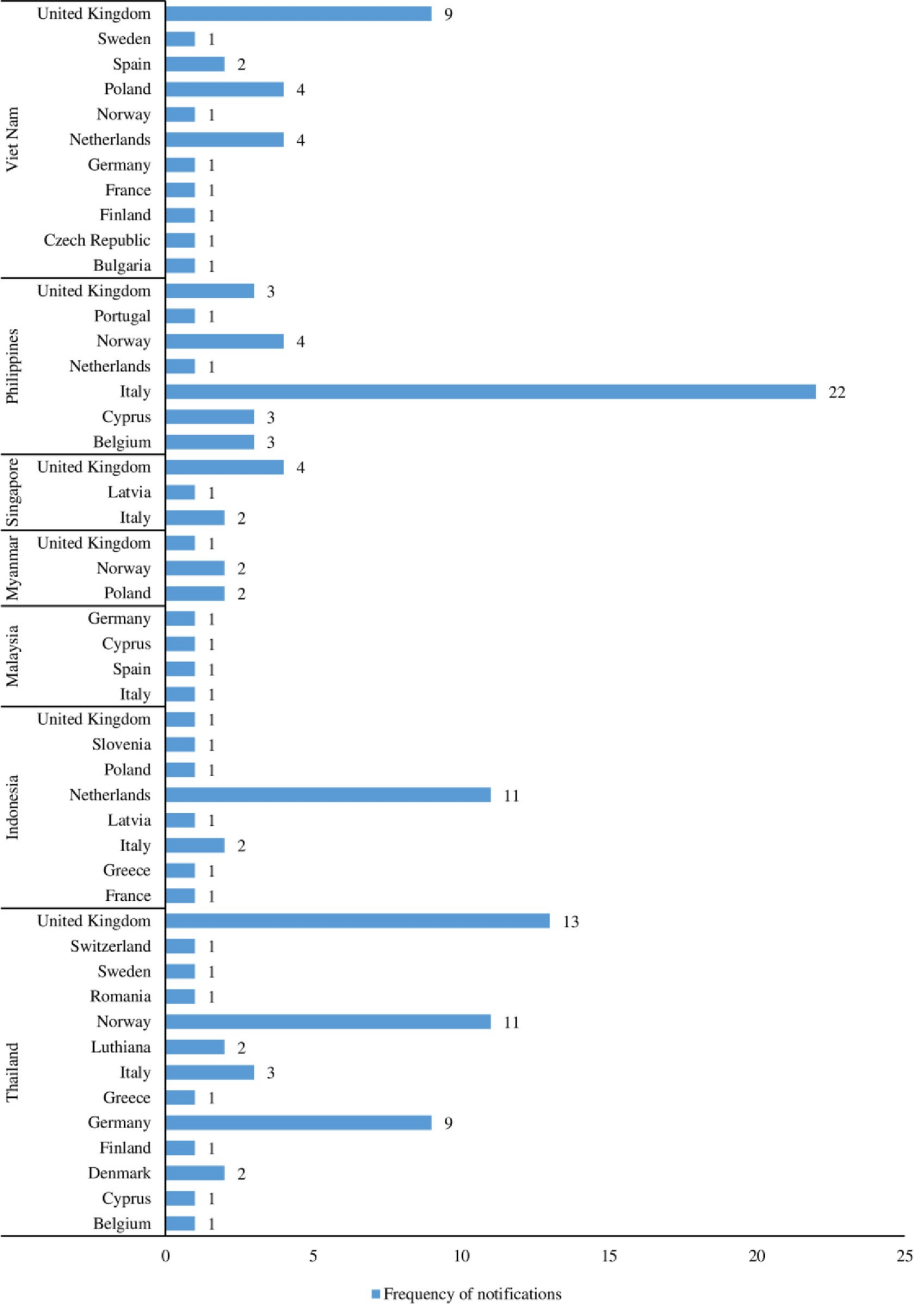
Frequency of notification s on food/feed exports from ASEA by importing countries (2000–2020).

Whereas other notifying countries as shown in Fig 8 possibly experienced least fraud on the exports as each of them recorded notification frequency; n < 10. Southeast Asia plays an increasingly essential role in the world food trade. In 2014, the ASEAN region become a net exporter of food, with approximately USD 139 billion in food exports when compared with USD 90 billion worth of food importations [28]. The proportion of agro-foods exported from within ASEAN has increased over the years, growing up from about 21% in 2000 to 29% in 2011. Afterwards, it has been diminishing to around 24% of the region’s sum of importations in 2014. Some countries have certain requirements which exporting countries find it difficult to fulfill.

Therefore, it would benefit the exporting countries to track down the right causes of FFA notifications on their exports to overcome these exportation difficulties. Thailand was reported to have received higher notifications for FFA on food/feed products exported to the United Kingdom, while exports from the Philippines had higher notifications from Italy when compared to other ASEAN countries. For Thailand and the Philippines most especially, knowledge and execution of the food safety standards of the importer including labelling and documentations were the major source of the problems. Furthermore, exports to these high-income countries were majorly processed foods (cocoa, cocoa preparations, coffee and tea; prepared dishes and snacks). These are highly valued products, and refusal to comply with their exceptional standards may amount to higher fraud notifications [29].

### Economic losses from ASEAN exports due to food fraud and adulterations

Due to the illicit economic incentives, food fraud, which is also referred to as economically motivated adulteration of food, happens all over the world and has been detected from many countries. According the [30] global trade of adulterated food could be worth about $50 billion. Although, premium food products are particularly prone to fraud and adulterations, food fraud has been observed in wide range of food products. However, the economic loss associated with FFA are difficult to quantify. The effect of economic losses due to FFA detected in exports are felt by both exporters and the industry. The financial losses could range from the destruction or detention of goods when detected at border of entry, loss to importer and food retailer from recall of food product, and litigations settlements when they are detected much later after consumption and possible health hazards. The values of seizure differs from case to case. For instance, in 2018, Eurojust, a European Union government agency charged with judicial cooperation among member state, with the help of Italian and Serbian national authorities uncover a large-scale transnational food fraud involving the use of decomposing apples, contaminated with toxic chemicals and mycotoxins, and refined with water and sugar to produce juice, jams, and other canned foods which were falsely labeled as organic products from European origin. The investigation led to the seizure of about 1,411 tonnes of products worth almost €5 million and illegal assets worth €6 million including the arrest of 9 suspects of the organized crime from both Italy and Serbia.

The market effect on the industry is also hard to measure. However, as stated earlier GMA puts the estimates for the cost of food fraud to global food industry to be between $10 billion and $15 billion annually. Food fraud and adulterations’ economic effect on industry has links to consumer trust and behavior towards the products. When food fraud is detected, it could influence consumer perception of specific brand, or all products from a particular country of origin which is the source of the fraud. It could also affect country of origin perception and impact other non-similar products from the same country. An empirical study by [31] examined the effect of media coverage of wine fraud on wine imported from different countries into China. Their study found that import demand for foreign wines especially wines from France were negatively affected in the short run by media coverage whenever wine fraud is detected. This often makes consumers, who are aware, to switch to other foreign brands or even local brands as distrust grows in short term. Study by [32] found that consumers’ willingness to pay premium for local brands of honey increases as they were informed of food fraud in imported honey and their potential health hazards. Many incidents of food fraud however go unnoticed, and it is impossible to know how pervasive the practice is. This is partly because most food fraud do not intend to pose food safety risk since they risk discovery and consumers do not often notice the quality problem. One of the common ways FFA are discovered is at importing country’s port of entry due to technologies that have been developed over the years and adopted by government to ensure food safety. Products that are fraudulent are often rejected at the border.

Exports from ASEAN have had its share of border rejections. The information on the values of food/feed product rejected at border are not available on EU RASFF database. Nonetheless, United Nations Industrial Development Organization [29] working paper in 2013 on regional trade standards compliance report (East Asia) presented some data that can give an estimate of the economic losses through border rejection from ASEAN countries [33]. The data were extracted and analyzed from EU RASFF, US OASIS. Australian Quarantine and Inspection Service (AQIS), and Japanese ministry of health, labor and welfare. Table 3 below presents the unit rejection per US$ billion of ASEAN food export by EU, US, Japan, and Australia. All eight of ASEAN members had combined 3,146 rejections per US billion-dollar in 2010 from EU, US, Japan, and Australia. Only about 9.58% of all border rejections to EU, US, Japan, and Australia in 2010 were for the reason of adulterations or missing documents. Therefore, it is estimated that in 2010, these eight ASEAN members had about 301 rejections per US billion-dollars export to these importing countries due to FFA. Moreover, many border rejections may have multiple reasons for rejection per time like in the case of fake organic food uncover by Eurojust described above. The fraud was related to false labelling of food as organic as well as the presence of mycotoxins in food.

**Table 3:** Unit border rejection of food and feed export per US$ billion of export value.

S/N	Country	Unit border rejection of food and feed export per US$ billion of export value to the following countries in 2010
		EU	US	Japan	Australia
1	Indonesia	6	142	40	215
2	Lao PDR	0	0	150	0
3	Malaysia	5	60	5	85
4	Myanmar	0	0	47	106
5	Philippines	8	162	15	1,111
6	Singapore	0	231	8	18
7	Thailand	36	75	108	30
8	Viet Nam	27	181	111	164

**Source**: UNIDO working paper 2013.

### Implication on food supply chain management

**Fig 9:**
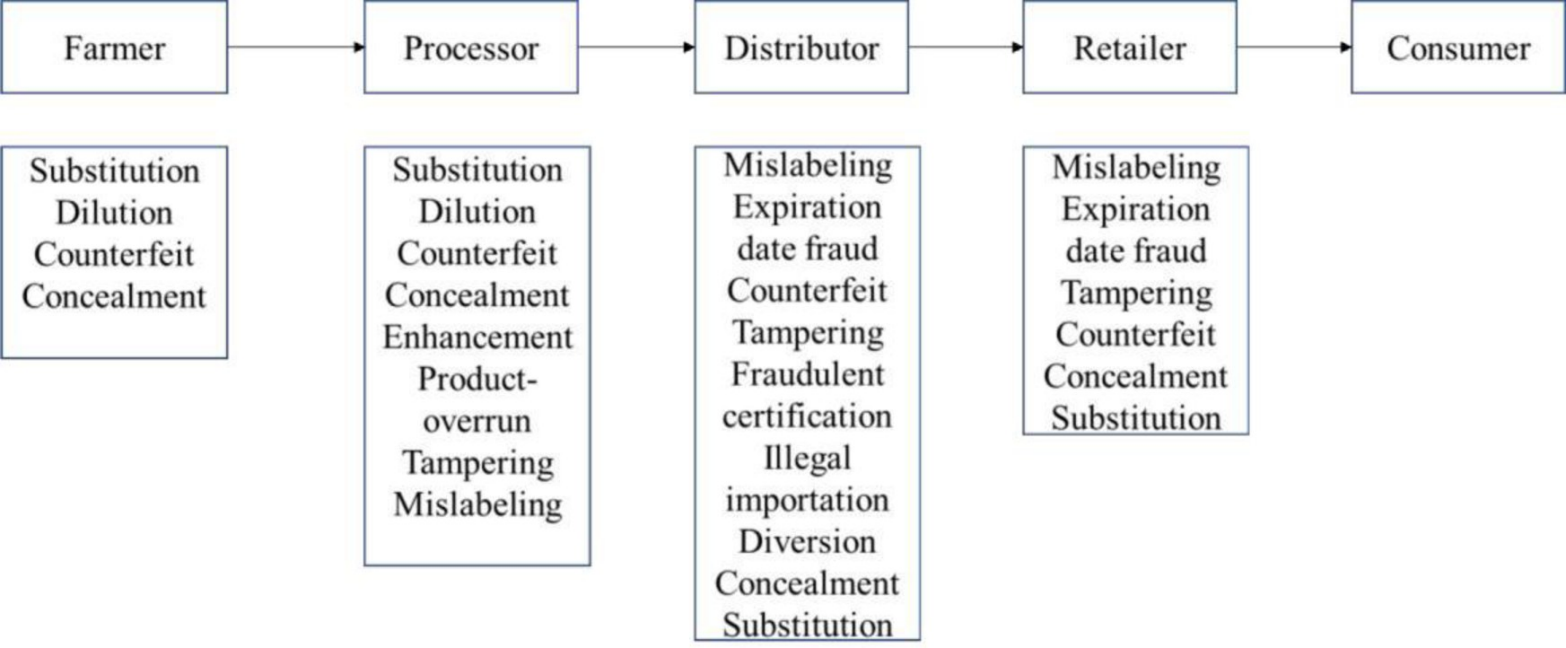
Food chain and possible FFA.

The root cause of FFA may come from the food supply chain through what is referred to as process integrity involving the activities undertaken to produce the product or it could come from distributional integrity which involves activities directed at misleading the origin of the food product. As an example of distributional integrity, in 2002 authorities in Australia detained a large shipment of honey which is labelled to have originated from Singapore, at which time Singapore was not producing honey. After investigation, it was discovered that the honey was from China and were illegally diverted from China to Singapore and then to Australia to avoid the ban on Chinese honey [34]. According to [35], fraud in food supply chain can arise from lack of product integrity, process integrity, people integrity, and data integrity. Product integrity refers to the quality characteristics of the product, process integrity entails activities performed in food processing to maintain the authenticity of the products, people integrity refers to the honesty and morals of people involved in food supply chain, and data integrity means the accuracy and consistency of information accompanying product throughout the food chain. The complexity and sub-contracting of production or processing of food products also provide opportunities for fraudulent activities by individuals in the supply chain. A typical food supply chain and possible types of fraud along the chain is presented in Fig 9 below. The rise in cases of FFA has made government institutions to implement stringent supply chain monitoring and certification procedures thus lengthening the process involved in international supply chain management. Various export, import, and inspection certificates are now required for many international trades to ensure food safety. It has also led to investment in costly equipment for product testing and traceability. Governments have also developed various food policies and programs to ensure food safety along the food supply chain.

In ASEAN, most of the member countries have established food safety regulations and standards that are in sequence with Codex Alimentarius standards. Countries in the region have increased the responsibilities of food business operators in ensuring food safety and many authorities are tasked with monitoring along the food chain. What may be lacking is the synergy and information sharing across various agencies and countries in the region. Several certifications are now developed such as good hygienic practices (GHP), good manufacturing practices (GMP), and enforcing sanitary and phytosanitary (SPS) measures as established by World Trade Organization (WTO) with increasing food inspection. Additionally, countries in the region now allocate tangible resources for inspection and have formulated guidance documents for hazard analysis and critical control points (HACCP), traceability, labelling, recall, food fraud vulnerability and mitigation. These various measures have the potential to increase consumer confidence.

## Conclusion and recommendation

It is evident that up till recent years, FFA are increasing threat in the agri-foods/feeds exported from ASEAN member states. This situation consequently impacts the industry economy including the confidence of the consumers. Exportations of adulterated herbs and spices without proper health certificates and full analytical reports especially from Indonesia; fish and fish products from Thailand and prepared dishes from the Philippines are main sources of concern in ASEAN food exports to the European countries. Generally, Thailand food products received the highest notifications on food/adulterations, occurring until year 2020. In addition, the rising stringent trade regulations may in turn add costs to the ASEAN stakeholders as most of the exported products that received FFA notification were rejected at the border and destroyed. Hence, this paper concludes with some policy recommendations that could help in improving international trade standards. Firstly, a more thorough implementation system is crucial to ensure compliance to trade standards. This very important because though farmers and producers are aware of the validity of trade certifications and standards, yet they are unwilling to comply due to ineffective incentive and monitoring structures. Programs and policies that support good manufacturing practices, sanitary standard operating procedure, awareness, and training for food/feed handlers should be encourage along food supply chain. Secondly, testing of food/feed products before exportation is generally an important factor in establishing confidence and relationship with buyers. In case the exporting countries are reluctant in sharing detailed information on their supply chain and processes, they should ensure that their own complete potential risk assessment and mitigation programs are adequately executed. Thirdly, by focusing on products that are prone to fraud and adulterations, factors that make such products vulnerable to FFA could be identified and resolved. Lastly, each ASEAN member states should create and constantly update their FFA database for annual systematic data analysis which could help to aid actions in curtailing future events.

## Supporting information

S1 Dataset Raw data from the European Union Rapid Alert System for Food and Feed (RASFF) extracted on fraud and adulterations notification on food/feed exports from seven ASEAN members (2000–2020). (XLSX)

S1 Fig Graphical abstract. (TIF)
