# Supplementary figures and images for: Incidence of fraud and adulterations in ASEAN food/feed exports: A 20-year analysis of RASFF’s notifications
Source: PLoS One. 2021 Nov 5;16(11):e0259298. doi: 10.1371/journal.pone.0259298 (PMC8570472; doi:10.1371/journal.pone.0259298)

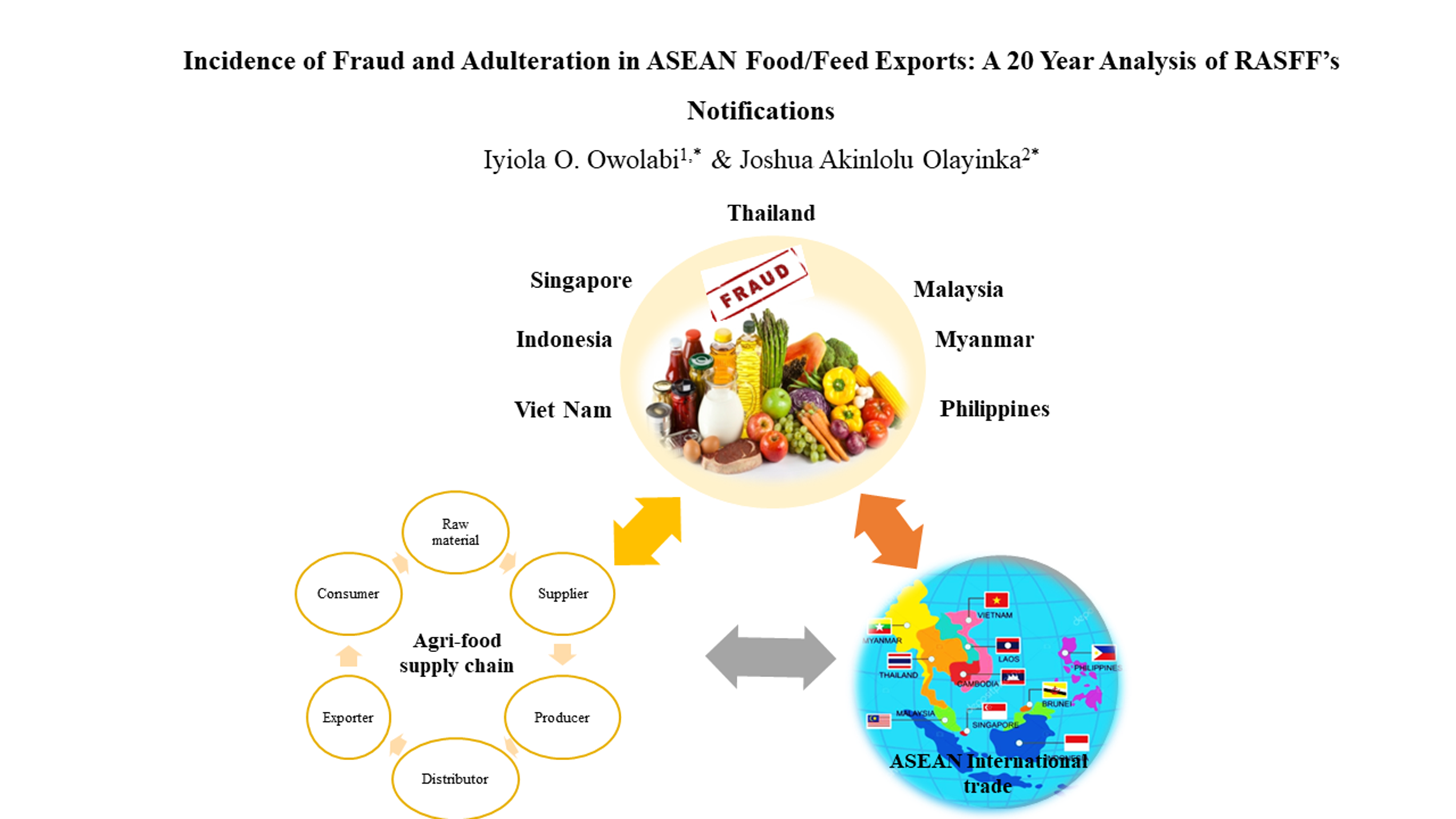

Supplement: S1 Fig — (TIF) [file pone.0259298.s002.tif]
